# Acupoint catgut embedding for chronic non-specific low back pain: A protocol of randomized controlled trial

**DOI:** 10.3389/fnins.2023.1106051

**Published:** 2023-02-01

**Authors:** Xiaohui Li, Xiuju Yin, Haiyan Feng, Wangbin Liao, Jiayou Zhao, Wu Su, Zhiyong Fan, Shan Wu

**Affiliations:** ^1^Guangdong Provincial Hospital of Chinese Medicine, The Second Affiliated Hospital of Guangzhou University of Chinese Medicine, Guangzhou, China; ^2^The Graduate School, Guangzhou University of Chinese Medicine, Guangzhou, Guangdong, China; ^3^Guangzhou Panyu Hospital of Chinese Medicine, Guangzhou, Guangdong, China

**Keywords:** chronic non-specific low back pain, acupoint catgut embedding, traditional Chinese medicine, randomized controlled trial, protocol, acupuncture

## Abstract

Chronic non-specific low back pain (CNLBP) is one of the leading causes of disability worldwide. Acupoint embedding (ACE) is widely used in China for the treatment of chronic non-specific low back pain, but there are no rigorous randomized controlled trials (RCTs) to confirm the effectiveness and safety of ACE for chronic non-specific low back pain. In this study, we design a single-center, single-blind, prospective RCT, with the aim of evaluating the efficacy and safety of ACE for CNLBP. 82 participants with CNLBP will be randomized in a 1:1 ratio into an ACE group and a sham ACE group. Participants will receive either ACE treatment or sham ACE treatment at once every 2 weeks, for an 8-week period, and followed by 6 months of follow-up. The primary outcome will be the change in visual analog scale (VAS) scores before and after treatment. Secondary outcomes will include the Oswestry Disability Index (ODI), the Roland Morris Disability Questionnaire (RMDQ) and the Short Form 36-Health Survey (SF-36). Adverse events that occur during the course of the trial will be recorded. Data will be analyzed according to a predefined statistical analysis plan. This study was approved by the medical ethics committee of Guangzhou Panyu Hospital of Chinese Medicine (202230). Written informed consent from patients is required. This trial is registered in the Chinese Clinical Trial Registry (ChiCTR2200059245). Trial results will be published in a peer-reviewed academic journal.

**Clinical trial registration:**

https://www.chictr.org.cn, identifier ChiCTR2200059245.

## 1. Introduction

Low back pain, a global high incidence and high burden disease, can greatly affect the quality of life of patients, and cause motor dysfunction or even disability (Knezevic et al., 2021). A systematic review of thirteen studies from Northern Europe, North America, and Israel reported that the prevalence of lower back pain ranged from 1.4 to 20.0%, with an annual incidence of 0.024 to 7%, and was highest in the United States (Fatoye et al., 2019). The incidence of chronic low back pain is expected to increase as the population ages, and as technological advances lead to increasingly sedentary lifestyles (Knezevic et al., 2021). A systematic analysis of the global burden of disease showed that low back pain increased the number of years lived with disability by 17.8% between 2007 and 2017 (Disease et al., 2018). A study comprehensively estimated the cost of care for non-severe low back pain episodes in hospitals in three Australian cities over a 5-year period, and showed that the average direct hospital cost for low back pain episodes was AUD$2959 (Coombs et al., 2021). It is estimated that more than $100 billion was spent annually on treating patients with low back pain in the United States (Katz, 2006). Most low back pain is non-specific (commonly cited as 90%) (Knezevic et al., 2021). Currently, for the management of patients with non-specific low back pain, pharmacological (non-steroidal anti-inflammatory drugs, weak opioids, and muscle relaxants) and non-pharmacological (exercise therapy, spinal manipulation, psychotherapy, and physiotherapy) treatments are recommended by guidelines (Vitoula et al., 2018). However, the treatment of patients with chronic non-specific low back pain (CNLBP) remains a great challenge. For example, oral non-steroidal anti-inflammatory drugs (NSAIDs) are recommended to be used with caution and not continuously, considering gastrointestinal and cardiovascular adverse events (Davis and Robson, 2016). Therefore, it is necessary to explore effective and safe non-pharmacological therapies.

Acupuncture, an important part of traditional Chinese medicine (TCM), has been proven to play a critical role in pain management and function recovery, especially for the treatment of CNLBP (Huang et al., 2021). Acupoint catgut embedding (ACE) is an innovative acupuncture method combined with traditional theory and modern materials, which embeds absorbed catgut into the acupoints, where the catgut will undergo softening, liquefaction, and absorption, thereby stimulating the acupoints for a long time (Teng et al., 2022). Compared with traditional acupuncture, ACE not only has the advantages of easy operation, strong stimulation, and long-lasting therapeutic effect, but also extends treatment interval patterns and reduces the discomfort caused by frequent acupuncture treatments, which can make up for the deficiencies in traditional acupuncture and improve patient compliance (Zhang et al., 2012). Previous preclinical studies have elucidated the mechanism of ACE in the treatment of pain. For example, in an *in vivo* experiment, ACE was shown to have a long-lasting analgesic effect on complete Freund’s adjuvant-induced inflammatory pain in rats, which was associated with activation of spinal 5-HT1AR, inhibition of GluN1 phosphorylation, and thus inhibition of Ca^2+^-dependent signaling (Cui et al., 2019). Another study suggested that ACE may exhibit antinociceptive effects by inhibiting Sig-1R regulation of p38 MAPK (Du et al., 2017). In recent years, a growing body of clinical evidence supports the use of ACE for the management of painful conditions. For example, a meta-analysis showed that ACE is beneficial for the relief of neck pain (Jo et al., 2022). However, to our knowledge, no clinical study on ACE for the treatment of CNLBP has been reported. Based on the current conditions, we will conduct a well-designed RCT to discuss the efficacy and safety of ACE, by comparing with a sham control in the treatment of CNLBP.

## 2. Methods

### 2.1. Design and setting

**FIGURE 1:**
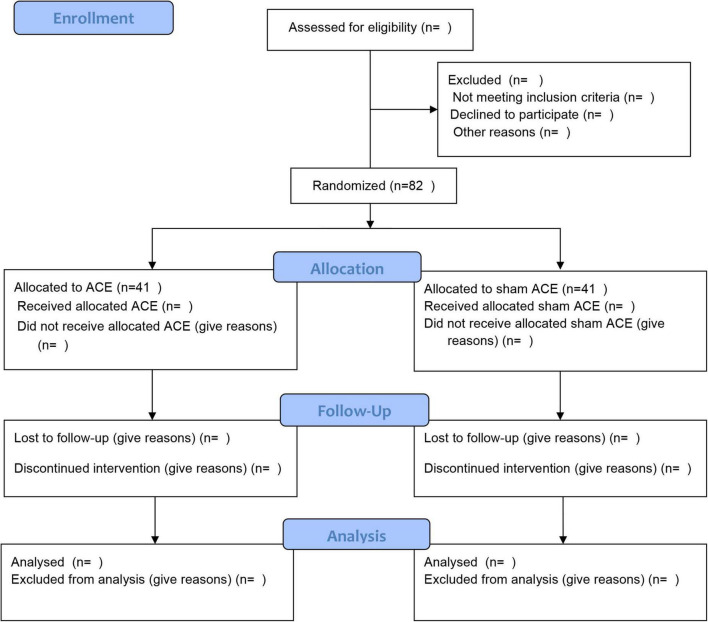
Flow chart of the study procedure.

The present study will be a prospective, randomized, patient-assessor-blinded, sham-controlled trial. A total of 82 participants will be recruited for the trial in Guangzhou Panyu Hospital of Chinese Medicine. Participants will be divided randomly at a ratio of 1:1 into the ACE group or the sham ACE group. The trial will contain an enrolment and allocation period of 3 days, an 8-week intervention period, and a 6-month follow-up period. The flow chart of the study procedure is shown in Figure 1. This study will be conducted in accordance with the Declaration of Helsinki, the Consolidated Standards of Reporting Trials (CONSORT) (Schulz et al., 2010) and the Standards for Reporting Interventions in Controlled Trials of Acupuncture (STRICTA) guidelines (MacPherson et al., 2010) in trial design and reporting. Any modifications to the protocol that may affect the conduct of the study will be expected to draft a formal modification of the protocol. Such update will be determined by the project management group and approved by the ethics committee, before the modification being placed into practice. This trial has been registered at the Chinese Clinical Trial Registry, numbered ChiCTR2200059245.

### 2.2. Recruitment

The recruitment advertisement for eligible participants will be placed on WeChat, official website of the hospital, and the waiting hall of the outpatient clinic, from April 2022 to December 2024. Eligible individuals who agree to participate in the study will sign written informed consents. By the way, participants will be notified that they are at liberty to withdraw from the study without any negative effects on their future treatments. After recruitment, the characteristic of participants will be preserved by dedicated personnel, which cannot be disclosed or used by unauthorized individuals at any time or for any reason.

### 2.3. Participants

#### 2.3.1. Diagnostic criteria

The diagnostic criteria for non-specific lower back pain are as follows: (1) pain or discomfort is located in the lumbar region with or without related radiation symptoms to lower extremities; (2) the history of low back pain lasts for more than 12 weeks; (3) the straight leg raising test is negative; and (4) there are no lumbar spine specific diseases diagnosed with imaging examination (e.g., presence of radiculopathy, osteoporosis, cord compression, or cancers). All potential participants will be first screened by a specialist with 20 years of clinical experience based on the diagnosis criteria.

#### 2.3.2. Inclusion criteria

Participants must meet with the following inclusion criteria: (1) 18–60 years old; (2) suffer from ongoing low back pain; (3) the visual analog scale (VAS) of low back pain intensity assessment is greater than 4 cm, but lower than 7 cm; and (4) participate voluntarily in this study, signing written informed consent. The inclusion process will be conducted by a specific researcher, after being assessed by diagnosis criteria.

#### 2.3.3. Exclusion criteria

Participants who meet any of the following conditions will be excluded: (1) cauda equina syndrome with bladder, bowel, or sexual dysfunction; (2) tumors, fractures, infections in the spine, or skin diseases in the lumbar region, or hemostatic disorder; (3) other serious diseases, such as cardiovascular, kidney, or liver diseases; (4) with diabetes or other diseases characterized by skin and subcutaneous tissue dysfunctions in absorption and renovation; (5) cognitive impairment or serious mental illnesses, such as schizophrenia or severe depression; (6) pregnant or breastfeeding women; (7) fear of acupuncture and have a history of severe acupuncture adverse reactions; (8) participation in other studies related to LBP treatment within the past 3 months; and (9) cannot participate in regular treatment or observation, as required. Exclusion process will be conducted by the same researcher handling the inclusion.

#### 2.3.4. Criteria for withdrawal, dropout, and removal

Participants presenting any of the following situations will be considered as the withdrawal cases: (1) the disease get worse after treatment in this study; (2) complications or severe adverse events (SAEs) occur during the trial; (3) the utilization of related treatments other than the study, which may impact upon the results of this study; (4) poor compliance, or the number of appointments for treatment does not meet the requirements (<80%); (5) unblinding for emergency situations; and (6) participants request to withdraw from the study.

Participants unable to complete the observation procedure of the study, regardless of the reasons, will be classified as dropout cases. The last outcome data of dropout cases will be included in data analysis. During the trial, participants will be removed if (1) they are wrongly enrolled due to misdiagnosis; (2) they do not accept the treatment after being enrolled in the study; (3) they participate in other clinical trials at the same time; and (4) the post-inclusion data is incomplete and there are no evaluable records for the analysis of the results. Data from withdrawal and dropout cases will be included in intention-to-treat analyses.

### 2.4. Randomization, allocation concealment, and blinding

Eligible participants will be divided randomly into the intervention group (ACE group) or the control group (sham ACE group). The random numbers of allocation sequences will be generated using Statistical Analysis System (version 9.4) by a statistician. Later, the random numbers and groupings will be packaged in opaque and closed envelopes with sequence numbers. The researchers responsible for the participant’s enrolment will take the envelopes with the corresponding number according to the inclusion order, and write down the name, gender, and age of the included participant on the surface of the envelope. All the marked envelopes will be kept by another researcher, who is not involved in participant recruitment.

After the baseline assessment of personal conditions and pre-treatment outcome measurements, acupuncturists will be informed about the different treatments of the participants. The outcome assessors will be blinded to the treatments due to the groups of the participants recorded in the case record forms (CRFs) being replaced by “A” and “B” without details. Additionally, the assessors will also be blinded to recruitment and groupings. All in all, participants, outcome assessors, and statistician in the study will be blinded to the different treatments. Under certain conditions, unblinding will be permissible when participants undergo SAEs or other emergency situations. Once unblinded, participants will withdraw from the trial, and the reasons will be documented and reported by the researchers.

### 2.5. Sample size

The VAS score is the primary outcome indicator for this trial. Based on the results of the pre-test, it is known that the VAS score in the control group was 4.64 ± 1.42 cm and is expected to be reduced by 1.02 cm after treatment with ACE, setting a two-sided test with α = 0.05, β = 0.10. A sample size of 34 cases per group was calculated using PASS 15 software. Considering the 20% lost to follow-up calculation, the final number of cases needed for at least two groups was 41, for a total of at least 82 participants included.

### 2.6. Interventions

The ACE operation in this study will be carried out in accordance with the Acupuncture Technical Operation Standard of the People’s Republic of China for ACE. Participants in both groups will accept treatments once every 2 weeks, for an 8-week period. During treatment, participants will be separated in different treatment rooms to avoid communication. Both treatments will be conducted by trained acupuncturists who are qualified, have acupuncture affiliation, and experience in clinical practice for more than 3 years. During the treatment and observation period of the study, if the participant’s pain symptoms worsen, the oral NSAIDs drug celecoxib (Pfizer Pharmaceuticals Ltd.) (Frampton and Keating, 2007; Foster et al., 2018) will be given orally for 2 days at a dose of 200 mg twice daily. If pain remains unrelieved, participants will be withdrawn from the study.

#### 2.6.1. Intervention group

**FIGURE 2:**
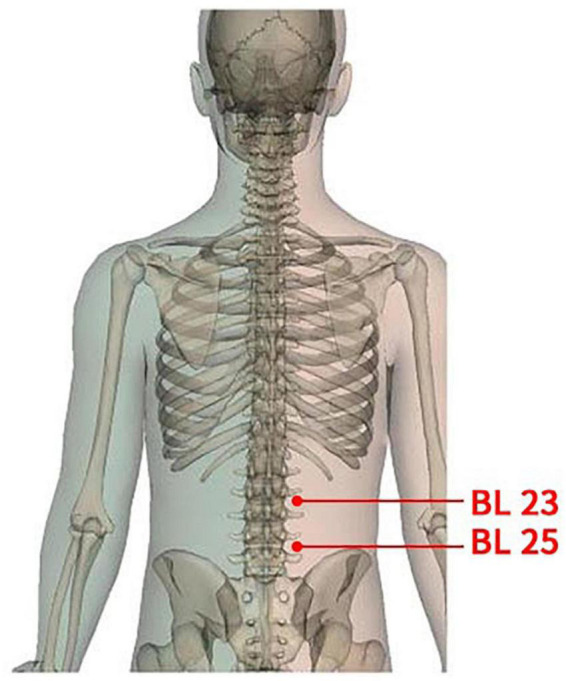
Locations of acupoints.

**FIGURE 3:**
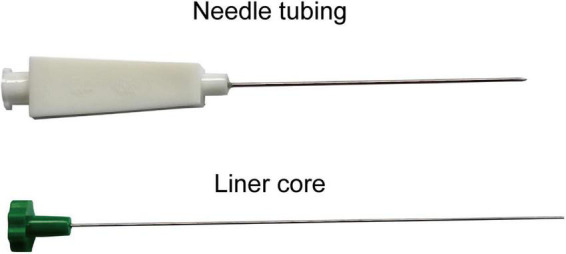
Parts of the acupoint catgut embedding device.

(1) **Acupoints:** According to the theory of TCM and previous clinical practice experience, this study will select the bilateral acupoints of Shenshu (BL23) and Dachangshu (BL25). The position and depth of acupoints for needle insertion are in accordance with the Name and Location of Acupoints (GB/T12346-2006), a standard of The People’s Republic of China. Details of selected acupoints are shown in Table 1 and Figure 2.
(2) **Preparation of ACE:** The main instruments utilized in the ACE treatment operation include an embedding needle, combinating of needle tubing and liner core, and absorbable surgical chromic catgut (Yangzhou Longhu Medical Instrument Co., Ltd., Yangzhou City, Jiangsu Province, China), all of which are disposable sterile products. The embedding needle is shown in Figure 3. Participants will stay in the prone position during the operation process.
(3) **Operation procedures:** Firstly, the skin of the target acupoint region will be exposed and sterilized with iodophor. After disinfecting hands and wearing sterile gloves, the acupuncturists will cut the absorbable surgical chromic catguts into 1 cm long pieces, and place the small catgut into the pinpoint of the embedding needle tubing, where the liner core will be moved up 1 cm. Then, acupuncturist will use the thumb and index fingers to fix the acupoint, while, with the other hand, inserting the needle perpendicularly to the skin surface of the acupoint at a depth of approximately 1 cm. Secondly, acupuncturists will apply lifting, inserting, and twisting techniques to stimulate special sensations, presenting soreness, such as numbness, swelling, or radiation, but no pain, which are described as *Deqi* in TCM theory. Thirdly, by pressing down the liner core of the embedding needle, the catgut in the needle tubing will be embedded in the muscle layer. Finally, both the liner core and needle tubing will be pulled out of the acupoint, and the post-treatment acupoint will be covered by a cotton ball with tape for 24 h.

**TABLE 1:** Locations of acupoints in the study.

	Acupoints	
	BL23 Bladder meridian	BL25 Bladder meridian
Needle insertion	On the lumbar region, on the same level of the second subspinous of the lumbar vertebra, and 1.5 cun lateral to the posterior midline.	On the lumbar region, on the same level of the fourth subspinous of the lumbar vertebra, and 1.5 cun lateral to the posterior midline.
Major indication and actions	Bone problems, low back pain, difficulty in urination, oedema	Pain in lumbar region and lower extremities.
Manipulation	Insert the needle 1.0 cm perpendicularly	Insert the needle 1.0 cm perpendicularly

For the standard and safe operation of ACE, the acupuncturists should: (1) ensure that the operation of ACE is conducted in a strictly sterile surrounding; (2) avoid catguts being embedded in the adipose tissue, which may cause fat liquefaction; (3) ensure that the chromic catgut is fully embedded in the deep tissue, and will not be exposed to the skin surface; (4) grasp at an appropriate angle and depth of the operation, to avoid damage to internal organs, major blood vessels, and nerves; and (5) inform participants regarding the precautions after treatment, such as keeping the lumbar region away from water for 24 h.

#### 2.6.2. Control group

In the sham ACE group, the embedding needle and acupoints are the same as those of the intervention group, but no catgut will be placed into the needle tubing before being inserted into the acupoints, so that no catgut will be left under the tissue after the needle is pulled out. At the same time, the needle will be withdrawn immediately, because inserting to the acupoints at a depth of 1.0 cm without retaining the needle to activate the *qi*, which is essentially different from traditional acupuncture.

### 2.7. Outcomes measurement

The efficacy evaluation in this study will include the primary outcome indicator VAS (Bijur et al., 2001). The secondary outcomes are the Roland-Morris Disability questionnaire (RMDQ) (Law et al., 2021), Oswestry Disability Index (ODI) (Mehra et al., 2008), and the Short Form 36-Health Survey (SF-36) (Taft et al., 2004). Safety outcomes will be evaluated by the incidence of adverse event. All evaluation indicators will be assessed at six time points: baseline, mid-treatment (4 weeks of treatment), post-treatment (8 weeks of treatment), and 1 month, 3 months, and 6 months after the treatments. Researchers who do not take responsibility for other processes in the study will be appointed as outcome assessors. Details of the observational items and the time window for data collection are shown in Table 2.

**TABLE 2:** Schedule of enrolment, interventions, and assessments.

	Enrolment and allocation		Treatment period				Follow-up period		
Timpepoint	Screening (−3 to 0 d)	Allocation (0 d)	2 w	4 w	6 w	8 w	1 m	3 m	6 m
**Enrollment**									
Vital signs	X		X	X	X	X			
Previous medical history	X								
Inclusion/exclusion assessment	X								
Informed consent	X								
Allocation		X							
**Interventions**									
ACE treatment		X	X	X	X	X			
sham ACE treatment		X	X	X	X	X			
**Assessments**									
VAS		X		X		X	X	X	X
ODI		X		X		X	X	X	X
RMDS		X		X		X	X	X	X
SF-36		X		X		X	X	X	X
Adverse events		X		X		X	X	X	X
Analysis of outcomes									X

d, day; w, week; m, month; ACE, acupoint catgut embedding; VAS, visual analog scale; RMDS, Roland Morris Disability Scale; ODI, Oswestry Disability Index; SF-36, the Short Form 36-Health Survey.

#### 2.7.1. Primary outcome

Visual analog scale will be used as the primary outcome to assess the intensity of CNLBP. Participants will be requested to measure their pain intensity on a 10-cm horizontal VAS, with 0 showing no pain and 10 showing the most severe pain.

#### 2.7.2. Secondary outcome

##### 2.7.2.1. The Roland-Morris Disability Questionnaire

Dysfunction of daily life caused by CNLBP will be assessed by the RMDQ, which contains 24 questions about the daily physical activities and function in daily life. Participants choose “yes” or “no” as the answer to each question, to describe their situations. The answer “yes” marks one score, with a total score ranging from 0 to 24, and the higher score reflecting more severe dysfunction.

##### 2.7.2.2. The Oswestry Disability Index

Oswestry Disability Index will be used to assess the disability in patients with CNLBP. The ODI consists of 10 items: pain, personal care, lifting, walking, sitting, standing, sleep, sex life, social life, and travel. The score for each question ranges from 0 to 5, according to the different degree from normal to severe. The total score will be worked out with a percentage, where the higher percentage means a more severe disability evaluation.

##### 2.7.2.3. The Short Form 36-Health Survey

Short Form 36-Health Survey is a general health measurement applied in populations with chronic diseases. It covers a systematic evaluation of physical and mental health with 8 domains (pain, physical functioning, role-physical, general health, vitality, role-emotional, mental health, and social functioning) covered by 36 questions. The score for each domain will be converted to a scale of 0–100, with higher scores indicating better quality of life.

#### 2.7.3. Safety assessment

Adverse events that happen during the study will be recorded in the CRFs in detail, including the time of occurrence, symptoms, duration, treatment methods, and outcomes. Adverse events will be assessed by the specialists in the panel, to determine whether the conditions are treatment related and whether further rescues are needed. Adverse events frequently occurring in ACE treatment include fainting, acupoint swelling, and subcutaneous hemorrhage, and will be addressed by symptomatic treatment methods. Once the adverse events are defined as severe, such as acupoints infection, allograft rejection, or major organ damage, the research process will be terminated and the inspectors of the monitoring panel will reveal the participant’s allocated intervention at once. Consequently, the participant will withdraw from the study and accept systemic treatments. The cost of treatments for adverse events arising from this study will be paid by the sponsor of this study. The monitoring panel will report the SAEs to the ethics committee, while the researchers will closely follow the participants’ condition, and record it in detail in the CRF.

### 2.8. Data collection and management

After the included participants sign the informed consent forms, researchers will collect individual information, such as general demographic information, clinical history, and baseline data for VAS, RMDQ, ODI, and SF-36. Outcome indicators will be measured at the previously indicated six time points, and documented in the CRFs. When the trial is completed, two researchers who are neither involved in the distribution nor the outcome evaluation will independently extract data from CRFs into an Excel table. The statistician will then conduct statistical analysis on this data after double check confirmation. To protect the privacy of participants, personal names will be replaced by numbers combined with initials. Researchers shall maintain data confidentiality for 5 years after the termination of the trial. In order to reduce the dropout rate of participants, researchers will contact the participants by telephone 2 days before their treatments to enquire on their conditions and encourage them to accept the treatments as scheduled.

### 2.9. Quality control

To achieve credibility and consistency in the study results, all researchers will receive standardized training in advance, including identification and allocation of eligible participants, treatment procedures, outcome evaluation, management of adverse events, and CRF completion. In addition, a data and safety monitoring panel consisting of three experts in different fields will be established, who are independent from the sponsor and have no conflict of interest with the study. The panel will conduct casual inspection to reduce potential biases in the study process and data analysis. Once any violation of the research requirements or SAEs are identified, the panel will notify the principal investigator, or suspend the study at any time.

### 2.10. Statistical analysis

Statistical analysis will be computed using Statistical Analysis System (SAS) v9.4 by a statistician. Continuous variables subject to normal distribution are expressed as mean ± standard deviation, otherwise they will be expressed as median (interquartile range). Discontinuous variables will be expressed as composition ratio and rate. The baseline comparison of continuous variables will be performed by *t*-test or non-parametric test, and the baseline comparison of discontinuous variables by the chi-squared test. Repeated measures analysis of variance (RM-ANOVA) will be performed on the measurement data at multiple observation time points, to analyze the trend over time and the interaction between treatment times. The statistical significance level will be 0.05 (bilateral) with 95% confidence interval. The safety analysis will mainly be based on descriptive statistics, including the incidence and the specific description of adverse events.

### 2.11. Dissemination

Results of the study will be presented at scientific conferences and in peer-reviewed publications. Participants included in the study will also have the opportunity to obtain the study results by telephone or e-mail.

### 2.12. Trial status

Currently, the protocol is version 1.0, registered on 27 April 2022. At the time of protocol submission, potential participants of the study have been actively enrolled.

## 3. Discussion

Chronic non-specific low back pain, one of the most common conditions in orthopaedics, rehabilitation, and pain medicine, accounts for 1/3 (Wang et al., 2015) of daily outpatient visits, and is a major cause of increase in years of life lived with disability, in both developed and developing countries (Diseases and Injuries, 2020). Recently, evidence has been published for ACE being beneficial in relieving pain from patients (Jo et al., 2022). However, the evidence for clinical studies targeting ACE in CNLBP treatment is lacking. This trial is a single-center, prospective, randomized, patient-assessor-blinded, sham-controlled trial conducted in China, to evaluate the clinical efficacy and safety of ACE for CNLBP patients, and provide new ideas for the management of CNLBP.

Pain, a major symptom plaguing patients with CNLBP, is a subjective experience that can be influenced by physical, psychological, personal experience, social and cultural factors (Wijma et al., 2016; Reis et al., 2022). An accurate and objective assessment of pain is essential to evaluate the efficacy of ACE. The VAS, a simple scale consisting of a 10-centimeter horizontal line, is widely used as a tool for measuring pain (Grilo et al., 2007). Moreover, one of the advantages of the VAS is that its value changes continuously. On the one hand, it reflects the subtle changes in pain, and, on the other hand, continuous scores can be used for statistic parameter testing, which is recognized internationally and superior to non-parameter testing of the category assessment scale. As a result, VAS will be applied as the primary outcome in this study. The ODI and RMDQ scores will be used to further evaluate the mobility disorders of patients with CNLBP, so as to obtain a more objective evaluation for the ACE treatment. In addition, the SF-36 will be used to assess participants’ quality of life. Appropriate acupoints selection is an essential factor for ACE treatment. We will select Shenshu (BL23) and Dachangshu (BL25), which are common acupoints for the treatment of low back pain in clinical practice. Both acupoints are located in the lumbar region, and can be used to treat disorders on or near this certain part of the body, according to the theory of meridian in TCM; further, a previous study indicated that BL23 and BL25 could decrease VAS and RMDQ scores for patients with low back pain (Wang, 2018).

However, there are several limitations in this trial. Firstly, this study is a single-center trial, and the study population is mainly from southern China, meaning that the applicability of ACE in other regions will be not discussed. Secondly, due to the different occupations or living habits of different participants, we do not impose strict requirements on the daily life of participants apart from the clinic, which may affect the results of the study. Finally, the trial lacks a positive control group, to distinguish superiority or inferiority relative to the available treatments. Despite these potential limitations, the results of this trial are expected to provide evidence for the efficacy and safety of ACE in the treatment of CNLBP, which will be useful to doctors, stakeholders, patients, and researchers.

## Ethics statement

The studies involving human participants were reviewed and approved by Guangzhou Panyu Hospital of Chinese Medicine (202230). The patients/participants provided their written informed consent to participate in this study.

## Author contributions

SW, ZF, and XL designed the study. XL and XY contributed equally to the study, conceptualized the study design, and wrote the manuscript. WL, JZ, and WS modified the manuscript. ZF, SW, HF, and XL participated in the modification of the study protocol. XL and HF designed the method for statistical analyses. All authors read and approved the final version of the manuscript.
